# Case Report: *SCN5A* mutations in three young patients with sick sinus syndrome

**DOI:** 10.3389/fcvm.2023.1294197

**Published:** 2023-12-01

**Authors:** Jiayu Liang, Suxin Luo, Bi Huang

**Affiliations:** Department of Cardiology, The First Affiliated Hospital of Chongqing Medical University, Chongqing, China

**Keywords:** sick sinus syndrome, gene mutation, *SCN5A* gene, gene testing, pacemaker implantation

## Abstract

**Background:**

Sick Sinus Syndrome (SSS) is generally regarded as a degenerative disease with aging; however, genetic mutations have been confirmed to be associated with SSS. Among them, mutations in *SCN5A* are common in patients with SSS. We report three young SSS patients with *SCN5A* mutations at different sites that have not been previously reported in Asian patients.

**Case presentation:**

The three patients were all young females who presented with symptoms of severe bradycardia and paroxysmal atrial flutter, for which two patients received ablation therapy. However, after ablation, Holter monitoring indicated a significant long cardiac arrest; therefore, the patients received pacemaker implantation. The three patients had familial SSS, and genetic testing was performed. Mutations were found in *SCN5A* at different sites in the three families. All three patients received pacemaker implantation, resulting in the symptoms of severe bradycardia disappearing.

**Conclusion:**

*SCN5A* heterozygous mutations are common among patients clinically affected by SSS. Their causative role is confirmed by our data and by the co-occurrence of genetic arrhythmias among our patients. Genetic testing for SSS cannot be performed as a single gene panel because of feasible literature results, but in presence of familial and personal history of SSS in association with arrhythmias can provide clinically useful information.

## Introduction

Sick Sinus Syndrome (SSS) is caused by impaired electrical automaticity of the sinus node or impaired conduction of electrical impulses generated by the sinus node to the surrounding atrial muscles, leading to sinus bradycardia, sinus block, or sinus arrest on the electrocardiogram (ECG) (1). Although SSS is usually regarded as a degenerative disease with aging, gene mutations have been confirmed to be associated with SSS in previous studies (2). Such genetic mutations include genes involved in sodium channels (3) and potassium channels (4), as well as *ANK2* (5), *HCN4* (6), and myosin heavy chain genes and their regulator genes (7), among which *SCN5A* (a sodium channel gene) is a common cause of familial SSS. Although SSS is prevalent in the elderly, patients with hereditary SSS can present with symptoms at a young age.

We report the cases of three unrelated young patients with familial SSS with missense mutations in *SCN5A*.

## Case presentation

### Case 1

**Figure 1:**
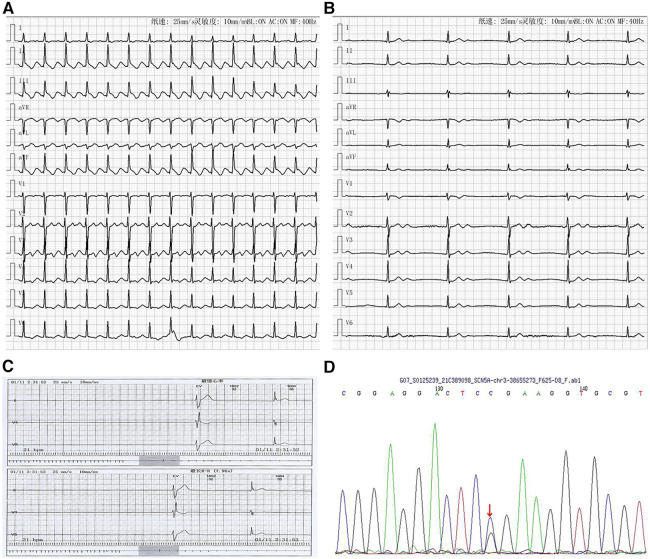
Arrhythmia in case 1. (**A**) Atrial flutter with 2:1 conduction; (**B**) Junctional escape rhythm with atrial standstill; (**C**) Holter indicated maximum R-R interval of 7.96 s; (**D**) Gene testing revealed SGN5A gene mutation (C.664C > G).

A 19-year-old Chinese woman was admitted to hospital due to left limb weakness. She has no history of cardiovascular disease but has occasional episodes of dizziness for 3 months. Her mother was diagnosed with SSS and received pacemaker implantation in her 30's. After admission, neurological examinations showed acute cerebral infarction in the right centrum semiovale and basal ganglia. At admission, ECG was obtained and it showed atrial flutter with 2:1 conduction (Figure 1A). The patient did not receive any pharmacological control for atrial flutter. One day later, the atrial flutter terminated spontaneously and it showed no obvious P wave and significant bradycardia with junctional rhythm (Figure 1B). A subsequent Holter examination indicated paroxysmal atrial fibrillation accompanied by a severe sinus arrest with a minimum ventricular rate of 21 beats per minute and the maximum R–R interval of 7.96 s (Figure 1C). Echocardiography revealed no structural and functional changes. In addition, cardiac magnetic resonance imaging did not display obvious fibrosis and other biochemical tests were all normal. Taken together, this young patient was diagnosed with SSS; her stroke was thought to be related to the atrial standstill and thrombosis. Subsequently, genetic testing (whole exome sequencing, Illumina NovaSeq 6,000, Illumina, US) indicated a heterozygous mutation from cytosine C to guanine G (C.664C > G) at nucleotide 664 of *SCN5A*, causing amino acid no. 222 to change from arginine to glycine (p.R222G) (Figure 1D). This mutation is consistent with her mother's genetic test results. Therefore, familial SSS was finally diagnosed, and this young patient received dual-chamber pacemaker implantation and prophylactic anticoagulation with edoxaban for stroke treatment. After pacemaker implantation, the sinus rate was around 55 beats per minute with 2.5% of atrial pacing by Holter monitoring. During a follow-up of 2 years, the patient had no symptoms of bradycardia and did not undergo stroke or other systemic embolisms.

### Case 2

**Figure 2:**
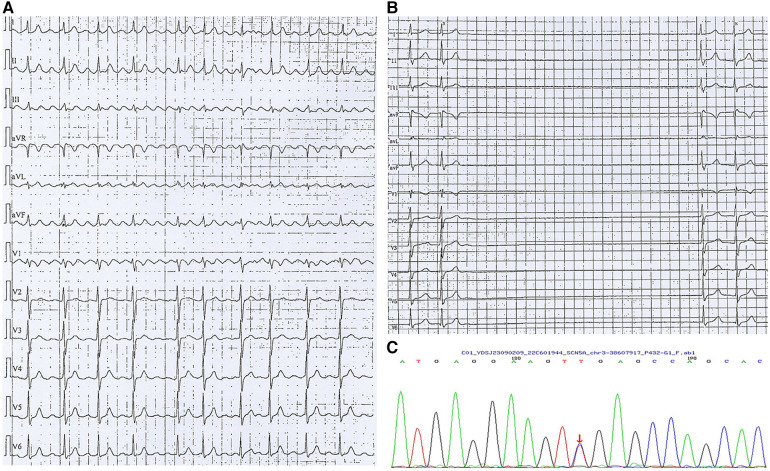
Arrhythmia in case 2. (**A**) Atrial flutter with 2:1 conduction; (**B**) Holter indicated maximum R-R interval of 6.73 s; (**C**) Gene testing revealed SGN5A gene mutation (c.3823G > A).

A woman aged 33 years was admitted to hospital due to paroxysmal palpitation lasting 5 years. She denied a history of cardiovascular disease and the physical examination was unremarkable. Her mother received pacemaker implantation in her 30's due to SSS. ECG upon admission showed atrial flutter (Figure 2A). Echocardiography and blood testing were normal. The patient was diagnosed lone atrial flutter and received radiofrequency ablation. However, after the procedure, Holter examination revealed that the minimum ventricular rate was 15 beats per minute and the maximum R–R interval was 6.73 s (Figure 2B). The severe bradyarrhythmia was sinus arrest and origined from atrial site or atrioventricular node. Therefore, SSS was diagnosed. Genetic testing (whole exome sequencing, Illumina NovaSeq 6,000, Illumina, US) revealed a heterozygous mutation in *SCN5A* located at nucleotide 3,823 from guanine G to adenine A (c.3823G > A), resulting in amino acid no. 1,275 changing from aspartic acid to asparagine (D1275N) (Figure 2C), consistent with her mother's genetic test results. The patient received dual-chamber pacemaker implantation and the sinus rate was around 60 beats per minute with 7.8% atrial pacing by Holter monitoring. Following pacemaker implantation, this patient no longer experienced palpitation.

### Case 3

**Figure 3:**
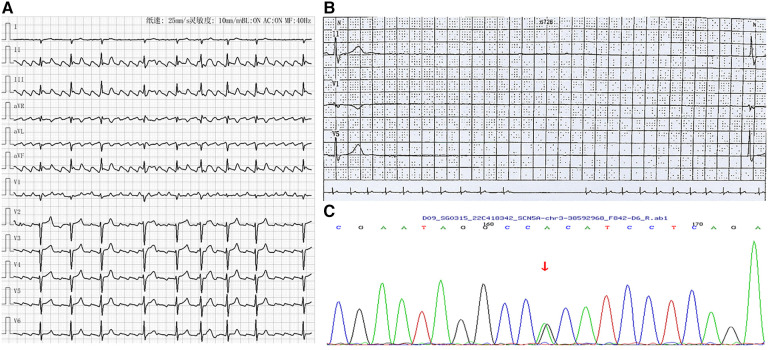
Arrhythmia in case 3. (**A**) Atrial flutter with 2-4:1 conduction; (**B**) Holter indicated maximum R-R interval of 4.11 s; (**C**) Gene testing revealed SGN5A gene mutation (c.4895G > A).

A woman in her 30s presented with recurrent dizziness and palpitation. She reported that she had experienced atrial flutter (Figure 3A) for 5 years without symptoms. Recently, she felt episodes of dizziness and palpitation and was admitted to hospital. Her mother also had atrial flutter in her 30's and received radiofrequency ablation; however, after the procedure, she presented with severe bradycardia and received pacemaker implantation. Due to the obvious symptom of atrial flutter, she requested radiofrequency ablation. However, severe bradycardia occurred after the procedure with a minimum ventricular rate of 32 beats per minute and a maximum R-R interval of 4.11 s by Holter monitoring (Figure 3B). She was also diagnosed with SSS and was recommended to receive pacemaker implantation; however, the patient refused. Genetic testing (whole exome sequencing, Illumina NovaSeq 6,000, Illumina, US) also demonstrated that she and her mother had the same heterozygous mutation in *SCN5A* located at position 4,895 from guanine G to adenine A (c.4895G > A), causing amino acid no.1,632 to be altered from arginine to histidine (p.R1632H) (Figure 3C). During the follow-up period, the patient was found to suffer from breast cancer and the maximum R-R interval by Holter monitoring was extended to 5.01 s. Finally, she received leadless pacemaker implantation and the sinus rate was around 50 beats per minute with 2.5% atrial pacing by Holter monitoring. During follow up, no symptoms of bradycardia occurred again.

**Figure 4:**
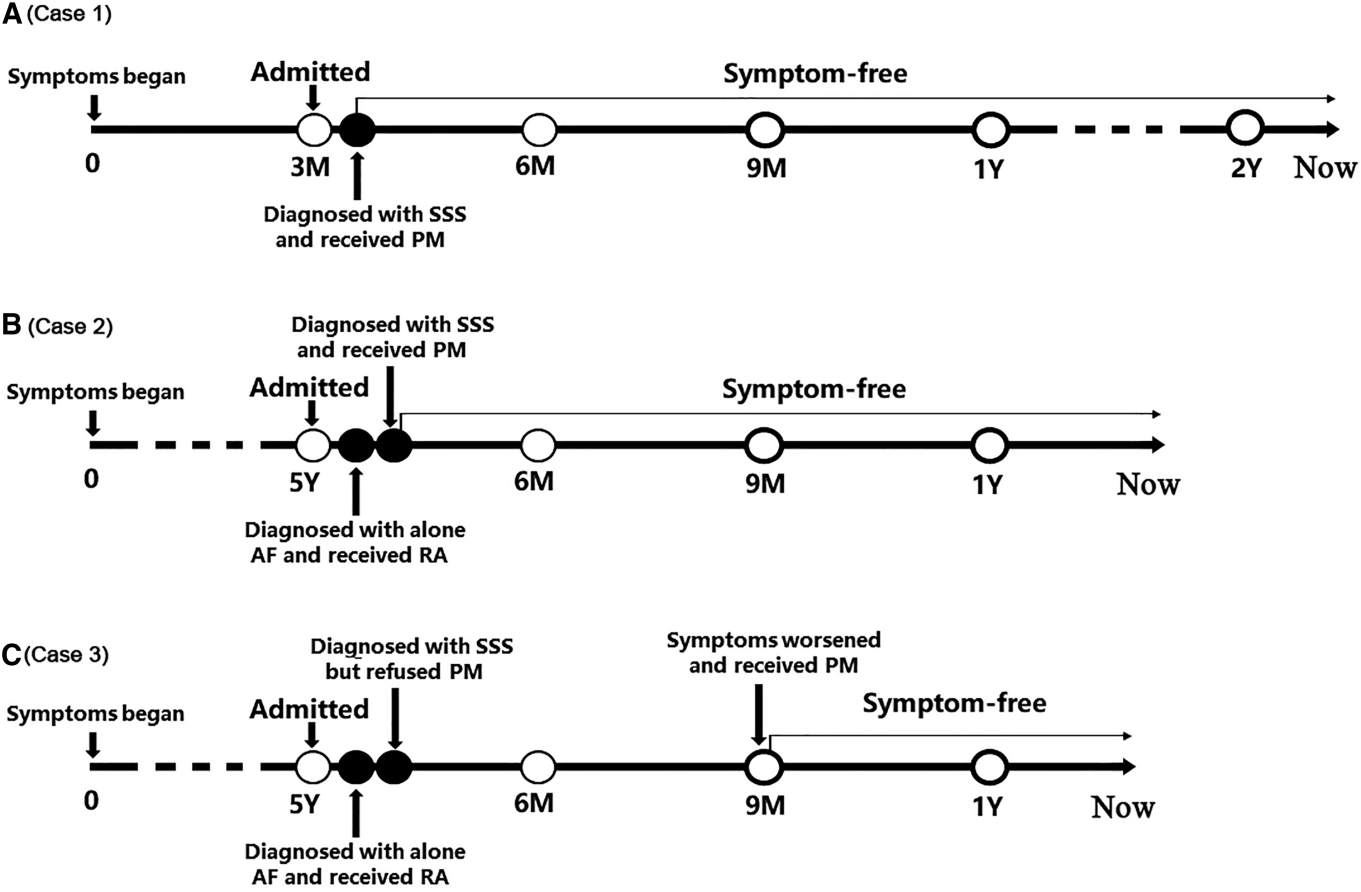
The timelines of the diagnosis and treatment in the three patients. (**A**) Timeline of case 1; (**B**) Timeline of case 2; (**C**) Timeline of case 3. AF, atrial flutter; PM, pacemaker; RA, radiofrequency ablation; SSS, sick sinus syndrome.

The timelines of the diagnosis and treatment in the three patients are shown in Figure 4. In Case 1 and Case 2, after diagnosed with SSS, they received pacemaker implantation immediately. In Case 3, the patient refused to receive pacemaker implantation when she was diagnosed with SSS; however, the symptoms worsened during follow up and finally she received pacemaker implantation. None of the three patients received any antiarrhythmic drug and neither underwent symptoms of bradycardia after pacemaker implantation.

## Discussion

In the present study, we report three unrelated young patients suffering from SSS, all of whom had a mutation in *SCN5A* at different sites. All patients received pacemaker implantation and no symptoms occurred again. Although SSS often develops in the elderly, it has been reported to occur at any age, including childhood. Therefore, SSS should be considered to be congenital due to the presence of gene mutations.

*SCN5A* is located in the first band of region 2 on the short arm of chromosome 3 (3p21) and encodes sodium channels in the cardiomyocytes, which plays a critical role in the excitation process underlying cardiac contraction (8). Previous studies have shown that mutations in *SCN5A* are related to a variety of hereditary arrhythmias, such as Brugada syndrome, long QT syndrome, familial progressive cardiac conduction disease (Lenegre disease), familial atrial fibrillation, and familial atrial standstill, etc. (9–13), among which Brugada syndrome is one of the most common phenotypes associated with single-copy *SCN5A* mutation (14–16). In fact, *SCN5A* mutation was the first genetic variant shown to be associated with Brugada syndrome (17) and has been shown to be responsible for 20%–25% of the disease in Caucasian populations (18). Mechanically, *SCN5A* mutations are associated with Nav1.5 loss-of-function either by decreased expression of Nav1.5 in the sarcolemma, expression of nonfunctional channels, or altered gating properties, resulting loss of action potential dome and consequently phase-2 re-entry upon arrival of a subendocardial action potential wavefront (19). Arrhythmias caused by *SCN5A* mutations can be divided into two categories according to the changes in sodium channel function; first is functionally enhanced mutations, such as long QT syndrome type 3, and the second is reduced function mutations, including Brugada syndrome, heart conduction system diseases, and SSS (20). Several reports have revealed the *SCN5A* mutant sites from the myocardium in humans (10, 21, 22); however, there appears to be no simple correspondence between mutated genes and phenotypes. One the one hand, patients who have the same mutation sites do not definitely present with the same clinical phenotypes; and on the other hand, mutations at the different sites may present the same arrhythmia. Therefore, there is a complex relationship between *SCN5A* mutations and the clinical phenotype.

Arrhythmias associated with *SCN5A* mutations have been previously reported to involve multiple mutant sites. The mutant sites in the three patients in the present study have also been reported previously. The first patient presented with C.664C > G (R222G) in *SCN5A* gene. Mutation in this site was reported to mainly relate with Purkinje ventricular arrhythmia and bigeminal arrhythmia possibly due to the missense amino acid substitution located in the S4 voltage sensor in domain I (23). However, Lehmann et al. (21) also reported a German family who suffered from atrial standstill and were found to have this site mutation in *SCN5A*. Further studies demonstrated that *SCN5A* mutation at this site reduced the current density with alteration of biophysical tissue properties, leading to SSS in an animal model (24). Although most studies reported this site mutation related with ventricular arrhythmia, bradyarrhythmia such as the atrial standstill is also a clinical phenotype, indicating a complex relationship between genotype and phenotype. Moreover, the patient's mother also diagnosed with SSS and received pacemaker implantation, and also had the same mutation site in *SCN5A* as in her daughter; however, the ECG of the patient's mother is unavailable and whether the clinical phenotypes were consistent between the patient and her mother deserves further study.

A previous study has shown that the mutation *SCN5A* (D1275N) is pathological (25); it was first reported by Groenewegen et al. (26) who found that co-inheritance of the mutation in *SCN5A* (D1275N) led to atrial standstill in a Dutch family, and it was associated with a range of cardiac diseases including dilated cardiomyopathy, sinus node dysfunction, atrial and ventricular tachyarrhythmias, conduction disease, etc. Subsequently the clinical phenotype associated with this mutation was extensively confirmed (22, 27–30). However, the clinical phenotype varies significantly, even if the mutation occurs at the same site, ranging from atrial to ventricular arrhythmia, from bradycardia to tachycardia, and from presenting in childhood to older age. Therefore, the mechanisms associated with the different clinical phenotypes are quite different. C.3823G > A makes negative charge of aspartic acid replaced by the neutral electric behavior of asparagine and therefore alters the electric properties of Nav1.5 (29). Furthermore, the clinical phenotypes are not completely isolated and have some overlap among the different clinical profiles. For example, case 2 in our study presented with familial atrial flutter and sinus pause, indicating the complexity in the pathogenesis of *SCN5A* (D1275N).

The *SCN5A* (R1632H) variant was reported in 2003; scholars from the United States recruited ten pediatric patients with an explicit diagnosis of congenital SSS from seven families. Probands of these patients exhibited six mutations in *SCN5A* identified by polymerase chain reaction, including the R1632H mutant (10). The *SCN5A* (R1632H) mutation was also found in the family of a 14-year-old girl, who described fast palpitations during exercise lasting a couple of minutes resulting in pre-syncope, heavy transpiration and retrosternal pain. After a series of examinations, she was diagnosed with atrial flutter and then received ablation (31). Studies have shown R1632H is related with various arrhythmias, including bradyarrhythmia, such as SSS, atrioventricular block, and tachyarrhythmia, such as Brugada syndrome. The clinical phenotype the present case 3 is similar to that reported in previous studies. Case 3 also received ablation, but presented with severe bradycardia after ablation, indicating that bradycardia–tachycardia syndrome is a common phenomenon in patients with *SCN5A* mutation. It also suggested that ablation should be carefully administered in young patients with bradycardia–tachycardia syndrome.

Notably, in addition to *SCN5A*, some genetic mutations such as in *MYH6* (32), *HCN4* (6), *PITX2*, *ZFHX3*, *TTN/CCDC141*, *SCN10A* and *KRT8* have been reported to associated with SSS (33); however, these genes above mentioned are included in the panel used in our present sequencing and did not detect any mutation, suggesting the mutation of *SCN5A* gene is responsible for the clinical phenotype in the three patients.

Our present study has some clinical implications. First, previous studies have shown familial SSS with *SCN5A* mutation has strong male predominance (34); however, in our present study, all three familial SSS patients were female, suggesting that both males and females are susceptible to *SCN5A* mutation. Second, although the three mutations have been reported previously, there is lack of Asian patients reported to suffer from these mutations. Our present study extends previous findings, indicating the clinical phenotypes associated with *SCN5A* mutation are similar among different races. Third, the frequency of these mutations in *SCN5A* such as C.3823G > A is unknown among population of Asian descent (35) and our present study provided some potential available data. In addition, although SSS is regarded as a degenerative disease and usually occurs in the elderly, taking our findings and previous studies together, patients with SSS associated with *SCN5A* mutation tend to have an earlier age of onset. When young patients present with SSS, there is a definite family history; thus, familial SSS should be considered and genetic testing is warranted.

There are some limitations in our present study. First, intracardiac maps during radiofrequency ablation could provide important information regarding the origin of atrial flutter and sinus rhythm as well as the electrophysiological characteristics of the conduction system. However, the intracardiac maps were unavailable in our present study. Second, some simple methods, such as current cardioversion can be used to evaluate the sinus function in patients with paroxysmal atrial arrhythmia. A severe bradyarrhythmia after cardioversion is an indication of sinus dysfunction and further radiofrequency ablation for such patients should be cautious. In our present study, none of the three patients undergo current cardioversion. However, if the patients received current cardioversion, it would probably present severe bradyarrhythmia due to the sinus node dysfunction and overdrive suppression by atrial flutter which was demonstrated in case 1. Third, the severe sinus node dysfunction in the three patients may be related with multigene variation in addition to *SCN5A* mutation, such as HCN and/or calcium channels. Silent variant in one of these channels combined with *SCN5A* mutation together could cause the severe sinus node dysfunction because previous studies have shown silent variation was involved in the pathogenicity of arrhythmia such as in long Q-T syndrome (36). Although we used whole exome sequencing to identify the potential gene variations and did not find mutated sites in HCN or calcium channels, the whole exome sequencing also has some limitations such as only identifying the common variations and may omit some rare or novel variations. Therefore, whole genome sequencing may provide more variation information and more studies are warranted. Last but not least, we only provided the clinical presentation and the genetic results, but did not provide evidence of mechanisms associated the mutation of *SCN5A* gene in our cases and the precise mechanisms needed to be clarified.

## Conclusion

*SCN5A* heterozygous mutations are common among patients clinically affected by SSS. Their causative role is confirmed by our data and by the co-occurrence of genetic arrhythmias among our patients. Genetic testing for SSS cannot be performed as a single gene panel because of feasible literature results, but in presence of familial and personal history of SSS in association with arrhythmias can provide clinically useful information.

## Data Availability

The original contributions presented in the study are included in the article/Supplementary Material, further inquiries can be directed to the corresponding authors.
